# Prevalence, risk factors, phenotypic and molecular characteristics for *Staphylococcus aureus* carriage in community-based drug users in Guangzhou, China

**DOI:** 10.1186/s13756-020-0698-9

**Published:** 2020-03-02

**Authors:** Yingying Wang, Jialing Lin, Junli Zhou, Zhigang Han, Zhenjiang Yao

**Affiliations:** 1 Department of Epidemiology and Health Statistics, Guangdong Pharmaceutical University, Guangzhou, 510310 China; 2 School of Public Health and Community Medicine, The University of New South Wales, Sydney, NSW Australia; 3 Department of AIDS/STD Control and Prevention, Guangzhou Center for Disease Control and Prevention, Guangzhou, 510310 China

**Keywords:** *S. aureus*, MRSA, Risk factor, Antimicrobial susceptibility, Drug user, Molecular characteristics

## Abstract

**Background:**

*Staphylococcus aureus* (*S. aureus*), particularly methicillin-resistant *Staphylococcus aureus* (MRSA), remains the predominant cause of infections in drug users. This cross-sectional study aims to elucidate the prevalence, risk factors, phenotypic and molecular characteristics of *S. aureus* carriage among community-based drug users.

**Methods:**

All eligible drug users, with both injection and non-injection route of drug administration, were asked to complete questionnaires and collect nasal swabs by trained personal during the period between May and December 2017 in Guangzhou, China. Swabs were processed for identification of *S. aureus*. Antimicrobial susceptibility test and polymerase chain reaction assays were used to detect phenotypic and molecular characteristics for identified isolates. Univariate and multivariate logistic regression analyses were used to assess risk factors for *S. aureus* carriage.

**Results:**

Overall, 353 drug users were included in the study and the prevalence of *S. aureus* carriage was 15.01% (53/353). The prevalence of MRSA carriage was 6.80% (24/353). Cohabitation was a risk factor for *S. aureus* (adjusted OR = 8.80, 95% CI: 1.89–40.99). The proportion of multidrug resistance was 54.72% for *S. aureus* isolates and most of these isolates were resistant to penicillin, erythromycin and clindamycin. Seventeen MRSA isolates were multidrug resistant. The results of clonal complexes (CCs) and sequence types (STs) for *S. aureus* were diverse. The three predominant types for CCs were CC5 (64.15%, 34/53), CC59 (11.32%, 6/53), and CC7 (7.55%, 4/53); and for STs were ST188 (20.75%, 11/53), ST5 (11.32%, 6/53), and ST59 (11.32%, 6/53).

**Conclusion:**

The prevalence of *S. aureus* nasal carriage was lower while the prevalence of MRSA carriage was moderate compared to previous studies. Phenotypic and molecular characteristics of *S. aureus* isolates, particularly MRSA isolates, revealed high proportions of antibiotic resistance, indicating the existence of cross-circulation, and implying high opportunity of virulence-related diseases. Decolonization and antibiotic stewardship might be implemented for drug users with MRSA carriage.

## Background

*Staphylococcus aureus* (*S. aureus*), particularly methicillin-resistant *S. aureus* (MRSA), continues to be a major pathogen in both hospital- and community-associated infections [1]. It has been reported that nasal carriers of *S. aureus* have an increased risk of being infected by this pathogen [2].

Based on the latest World Drug Report, an estimated 271 million people aged 15–64 used drugs, with both injection and non-injection route of drug administration and 35 million people were estimated to be suffering from drug use disorders in 2017 [3]. Obviously, illicit drug use is a global public health problem. In recent studies, the prevalence of *S. aureus*, particularly MRSA carriage, among drug users is higher compared to the general population [4, 5]. The phenotypic and molecular characteristics of *S. aureus* isolates in drug users were little reported. Most of these studies were conducted in developed countries, including the United States of America, Canada, and European countries. There is no similar work conducted among drug users in China.

According to the above facts, it is necessary to investigate the epidemiology of *S. aureus* carriage, particularly MRSA carriage, among drug users, in China. Therefore, in this study, we aimed to elucidate the prevalence, risk factors, phenotypic and molecular characteristics of *S. aureus* from the nasal cavity of community-based drug users in Guangzhou, China.

## Methods

### Ethics statement

The study was approved by the Ethics Committee of Guangdong Pharmaceutical University, and it was performed in accordance with the approved guidelines. Written informed consent were obtained from all participants.

### Study design and participants

A cross-sectional study of *S. aureus* nasal carriage among all drug users, with both injection and non-injection route of drug administration, was conducted between May and December 2017 in three community health service centers, Guangzhou, China. Participants who had used drug in the previous 12 months were voluntarily recruited in the study. Drugs included opiates, heroin, methamphetamine (methamphetamine), morphine, marijuana, cocaine, and other addictive narcotic drugs and psychotropic substances. Those participants with psychiatric illness or acute diseases were excluded. A face-to-face questionnaire was used to collect relevant information, including demographics (age, sex), socio-related characteristics (employment status, living conditions, income levels, history of homelessness, and history of incarceration), behavior (history of sex and the number of sexual partners), health-related characteristics (human immunodeficiency virus (HIV) status, hepatitis, antibiotic use, skin infection, hospitalization, and history of needle exchange), and periods, and route of drug use. In this study, cohabitation refers to someone living together with another person without marriage.

### Isolation and identification of *S. aureus*

After completing the questionnaire part of the study, trained personnel collected swabs from both anterior nares of the participants. The swabs were soaked in 7.5% sodium chloride broth at 4 °C during transportation, and then incubated at 37 ± 1 °C for 24 h for further experiments. The swabs were used to inoculate mannitol salt agar for 24–48 h incubation. Samples were identified as *S. aureus* isolates when they had specific colony morphology and were positive for gram staining, catalase reaction, hemolysis test, DNase test, coagulase tests, and *16S rRNA* and *nuc* genes. Two colonies were picked from one mannitol plate. Those *S. aureus* isolates that were resistant to cefoxitin and/or positive for *mecA* gene were identified as MRSA isolates, all other *S. aureus* isolates were identified as methicillin-sensitive *S. aureus* (MSSA). More details were described in the previous work [6].

### Phenotypic characterization

The antimicrobial susceptibility of all *S. aureus* isolates was determined by the disk diffusion method, following the guidelines of the Clinical and Laboratory Standards Institute of 2015. The following antibiotics were tested: clindamycin, erythromycin, penicillin, linezolid, gentamicin, teicoplanin, moxifloxacin, trimethoprim-sulfamethoxazole, rifampin, chloramphenicol, and tetracycline. The reference *S. aureus* strain ATCC 25923 and ATCC 29213 were used for quality and positive control. We classified the isolates as susceptible and resistant to each antibiotic. Those isolates resistant to ≥1 agent in ≥3 antimicrobial categories were identified as multidrug resistant (MDR) [7]. More details were described in previous work [6].

### Molecular characterization

All *S. aureus* isolates were also tested for the carriage of tetracycline-resistant genes [*tet*(M), *tet*(K)] and erythromycin-resistant genes [*erm*(A), *erm*(C)]. All *S. aureus* isolates were further tested to confirm the presence of toxin genes including Panton-Valentine leukocidin genes (*lukF-PV* and *lukS-PV*), Toxic shock syndrome toxin-1 gene (*tst*), Exfoliative toxin A gene (*eta*), Exfoliative toxin B gene (*etb*) and Staphylococcal enterotoxins (SEs) (*sea*-*see*, *seg*-*ser*, *seu*) genes. Multilocus sequence typing (MLST) was performed to confirm clonal complexes (CCs) and sequence types (STs). Additionally, all MRSA isolates were tested for Staphylococcal cassette chromosome *mec* (SCC*mec*) typing. More details were described in previous work [6].

### Statistical analysis

The data were entered using Epidata 3.1 (EpiData Associa-tion, Odense Denmark) and exported to Stata 14.2 (College Station, Texas, USA) software for further analysis. We assessed the associations between *S. aureus* carriers and relevant characteristics by the following methods. Univariate analyses were conducted using the Pearson’s chi-squared test or the Fisher’s exact test when appropriate. Multivariate logistic regression models were used to determine risk factors associated with *S. aureus* carriage. Independent risk factors with a *P* < 0.1 in univariable logistic regression analysis were included in the multivariable models. Potential confounding covariates were adjusted in the models. A two-sided *P*-value of ≤0.05 was defined as statistical significance.

## Results

### Prevalence of *S. aureus* carriage

A total of 353 drug users were eligible for inclusion in the study. The prevalence of *S. aureus* nasal carriage in drug users was 15.01% (53/353). The prevalence of MRSA carriage was 6.80% (24/353).

### Risk factors of *S. aureus* carriage

**Fig. 1:**
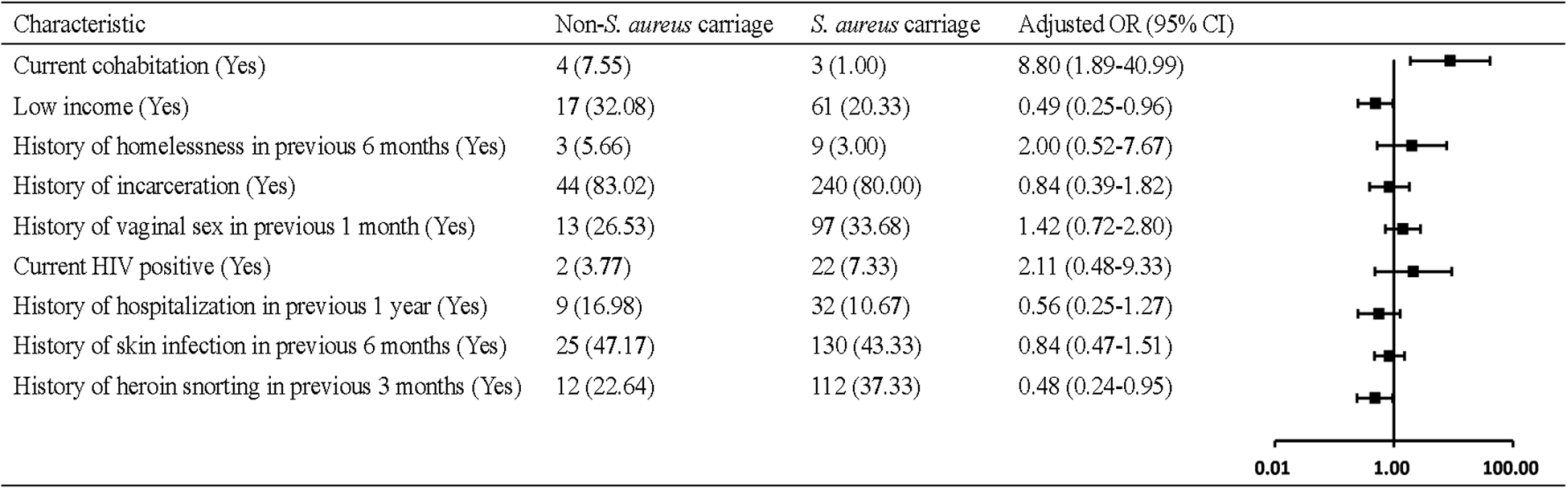
Multivariate analysis of risk factors for *S. aureus* carriage among community-based drug users in Guangzhou, China, 2017. *S. aureus*, *Staphylococcus aureus*; No., Number of; OR, Odds ratio; CI, Confidence interval; HIV, Human immunodeficiency virus

Table 1 shows univariate analyses of *S. aureus* carriage among drug users. After adjusting for confounding covariaes, current cohabitation was still a risk factor for *S. aureus* carriage (aOR = 8.80, 95% CI: 1.89–40.99) in drug users (Fig. 1).

**Table 1:** Univariate analysis of risk factor for *S. aureus* carriage among drug users in Guangzhou, China, 2017

Characteristics	Non-*S. aureus* carriage (*N* = 300)	*S. aureus* carriage (*N* = 53)	*P*-value
Demographics-level			
Sex (Male)	262 (87.33)	47 (88.68)	0.784
age (> 50)	169 (56.33)	26 (49.06)	0.326
Social-level			
Current employed (Yes)	80 (26.67)	14 (26.42)	0.970
Current cohabitataion (Yes)	3 (1.00)	4 (7.55)	0.011
Low income (Yes)	61 (20.33)	17 (32.08)	0.058
History of homelessness in past 6 months (Yes)	9 (3.00)	3 (5.66)	0.400
History of incarceration (Yes)	240 (80.00)	44 (83.02)	0.609
Behavior-level			
History of vaginal sex in past 1 month (Yes)	97 (33.68)	13 (26.53)	0.324
Number of sexual partners in past 1 year (> 1)	28 (9.33)	2 (3.77)	0.283
Health-level			
Current HIV positive (Yes)	22 (7.33)	2 (3.77)	0.553
Current hepatitis (Yes)	114 (38.00)	24 (45.28)	0.316
Antibiotic use in past 6 months (Yes)	83 (27.67)	16 (30.19)	0.706
History of hospitalization in past 1 year (Yes)	32 (10.67)	9 (16.98)	0.186
History of skin infection in past 6 months (Yes)	130 (43.33)	25 (47.17)	0.604
History of needle exchange in past 1 year (Yes)	26 (8.67)	4 (7.55)	1.000
Drug use-level			
Period of drug use (> 10 years)	18 (6.00)	0 (0.00)	0.087
History of heroin snorting in past 3 months (Yes)	188 (62.67)	41 (77.36)	0.039
History of intravenous heroin in past 3 months (Yes)	113 (37.67)	14 (26.42)	0.116
History of using injection drugs in past 3 months (Yes)	187 (62.33)	39 (73.58)	0.116

*S. aureus Staphylococcus aureus*; *N* Number of total participants; *HIV* Human immunodeficiency virus

### Phenotypic characteristics

**Fig. 2:**
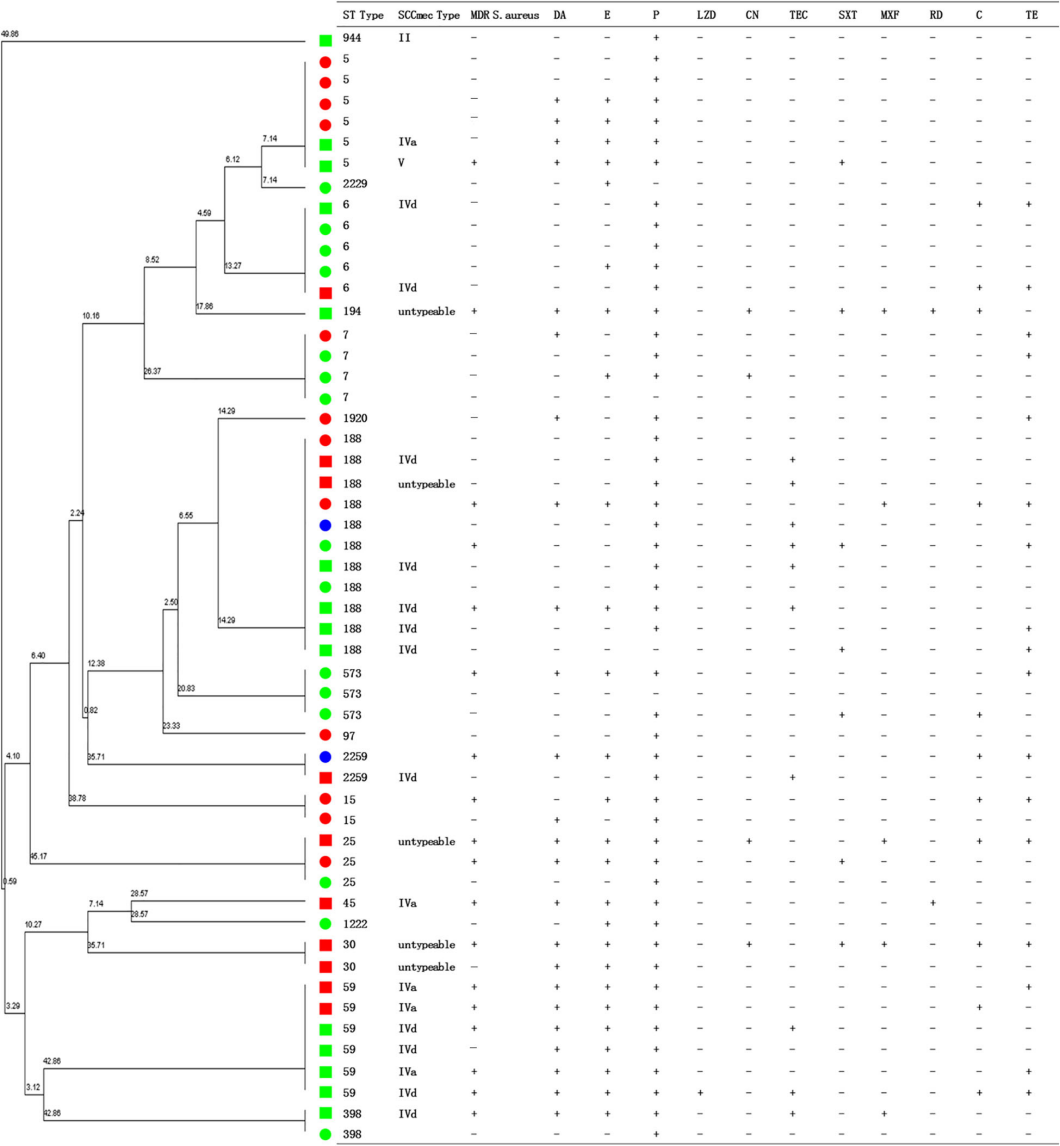
Clonal dendrogram and detailed information of *S. aureus* isolates for community-based drug users in Guangzhou, China, 2017. Isolates with the same color represented they were from the same community; squares represented MRSA isolates and circles represented MSSA isolates. *S. aureus*, *Staphylococcus aureus*; MRSA, Methicillin-resistant *S. aureus*; MSSA, Methicillin- sensitive *S. aureus*; ST, Sequence type; SCC*mec*, Staphylococcal cassette chromosome *mec*; MDR, Multidrug resistance; P, Penicillin; E, Erythromycin; DA, Clindamycin; TE, Tetracycline; C, Chloramphenicol; SXT, Trimethoprim-sulfamethoxazole; TEC, Teicoplanin; MXF, Moxifloxacin; CN, Gentamicin; RD, Rifampin; LZD, Linezolid

The antibiotic susceptibility testing results revealed that most *S. aureus* isolates were susceptible to linezolid, rifampin and gentamicin, but resistant to penicillin (92.45%), erythromycin (49.06%), clindamycin (45.28%) and tetracycline (32.08%) (Table 2). Eighteen *S. aureus* isolates were MDR. Notably, 38.89% of MDR *S. aureus* were resistant to erythromycin, clindamycin and chloramphenicol. For MRSA isolates, the proportion of MDR MRSA was 36.00% (9/24) (Fig. 2). The proportions of antibiotic resistance were higher in MRSA isolates than MSSA isolates (Table 2).

**Table 2:** Phenotypic and molecular characteristics of *S. aureus* isolates among drug users in Guangzhou, China, 2017

Characteristics	*S. aureus* (*N* = 53)		
	Total	MRSA(*N* = 24)	MSSA (*N* = 29)
Resistant phenotype (resistant)			
Clindamycin	24 (45.28)	15 (62.50)	9 (31.03)
Erythromycin	26 (49.06)	15 (62.50)	11 (37.93)
Penicillin	49 (92.45)	23 (95.83)	26 (89.66)
Linezolid	1 (1.89)	1 (4.17)	0 (0.00)
Gentamicin	4 (7.55)	3 (12.50)	1 (3.45)
Teicoplanin	10 (18.87)	8 (33.33)	2 (6.90)
Trimethoprim-sulfamethoxazole	7 (13.21)	4 (16.67)	3 (10.34)
Moxifloxacin	5 (9.43)	4 (16.67)	1 (3.45)
Rifampin	2 (3.77)	2 (8.33)	0 (0.00)
Chloramphenicol	11 (20.75)	7 (29.17)	4 (13.79)
Tetracycline	17 (32.08)	9 (37.50)	8 (27.59)
Resistant genotype (positive)			
*erm*(A)	1 (1.89)	1 (4.17)	0 (0.00)
*erm*(C)	5 (9.43)	5 (20.83)	0 (0.00)
*tet*(K)	4 (7.55)	4 (16.67)	0 (0.00)
*tet*(M)	0 (0.00)	0 (0.00)	0 (0.00)
Virulence genes (positive)			
*lukF-PV and lukS-PV*	3 (5.66)	3 (12.50)	0 (0.00)
*tst*	2 (3.77)	2 (8.33)	0 (0.00)
*eta*	1 (1.89)	1 (4.17)	0 (0.00)
*etb*	1 (1.89)	1 (4.17)	0 (0.00)
*sea*	0 (0.00)	0 (0.00)	0 (0.00)
*seb*	1 (1.89)	0 (0.00)	1 (3.45)
*sec*	3 (5.66)	1 (4.17)	2 (6.90)
*sed*	17 (32.08)	11 (45.83)	6 (20.69)
*see*	0 (0.00)	0 (0.00)	0 (0.00)
*seg*	26 (49.06)	13 (54.17)	13 (44.83)
*seh*	3 (3.45)	3 (12.50)	0 (0.00)
*sei*	18 (33.96)	9 (37.50)	9 (31.03)
*sej*	6 (11.32)	3 (12.50)	3 (10.34)
*sek*	11 (20.75)	8 (33.33)	3 (10.34)
*sel*	4 (7.55)	2 (8.33)	2 (6.90)
*sem*	16 (30.19)	6 (25.00)	10 (34.48)
*sen*	12 (22.64)	4 (16.67)	8 (27.59)
*seo*	13 (24.53)	4 (16.67)	9 (31.03)
*sep*	4 (7.55)	1 (4.17)	3 (10.34)
*seq*	5 (9.43)	2 (8.33)	3 (10.34)
*ser*	5 (9.43)	2 (8.33)	3 (10.34)
*seu*	12 (22.64)	7 (29.17)	5 (17.24)

*S. aureus Staphylococcus aureus*; *MRSA* Methicillin-resistant *S. aureus*; *MSSA* Methicillin- sensitive *S. aureus*

In terms of macrolide-resistant genes, five (9.43%) *S. aureus* isolates were positive for the *erm*(C) and one (1.89%) was positive for the *erm*(A) gene. Only one *S. aureus* isolate was positive for both the *erm*(C) and *erm*(A) genes. For tetracycline-resistant genes, four (7.55%) *S. aureus* isolates were positive for the *tet*(K) and no isolate was positive for *tet*(M) gene. Additionally, only one *S. aureus* isolate was positive for *erm*(C), *erm*(A) and *tet*(K) genes. These gene-positive *S. aureus* isolates were all MRSA isolates.

### Molecular characteristics

Overall, 8 CCs and 18 STs were detected from 53 *S. aureus* isolates (Fig. 2). Three of the most predominant CCs were CC5 (34), CC59 (6), and CC7 (4). Three of the most predominant STs were ST188 (11), ST5 (6), and ST59 (6). For 24 MRSA isolates, 7 CCs and 13 STs were detected. Two of the most predominant CCs were CC5 (13) and CC59 (6). Two of the most predominant STs were ST188 (6) and ST59 (6).

In terms of virulence genes (Table 2), 5.66% of *S. aureus* isolates were positive to *lukF-PV and lukS-PV* genes. Two MRSA isolates were positive to the *tst* gene. Notably, only one MRSA isolate was positive to the *eta* gene and one to the *etb* gene. For the SEs genes, the three most predominant genes were *seg* (49.06%), *sei* (34.96%) and *sad* (32.08%). All *S. aureus* isolates were negative to the *sea* and *see* genes.

A total of four SCC*mec* types were detected from the 24 MRSA isolates, in which 12 isolates were type IVd, five were type IVa, one was type V, one was type II, and five were non-typeable (Fig. 2).

## Discussion

To the best of our knowledge, this is a relatively comprehensive study which contributes to the understanding of the prevalence, risk factors, phenotypic and molecular characteristics for *S. aureus* nasal carriage among community-based drug users in China. The prevalence of *S. aureus* carriage in the study (15.01%) is lower than previously reported estimates which ranged from 19.79 to 45.05% [4, 8–11]. Participants of those previous studies were injection drug users. In this study, however, only 64.02% of participants had history of using injection drugs in the past 3 months. Additionally, we found that a majority of long-term drug users who took drugs by snorting had few vibrissae. This might also be a potential factor leading to a low prevalence of *S. aureus* carriage, further studies about the impact of snorting drugs on *S. aureus* carriage need to be conducted in the future The prevalence of MRSA nasal carriage (6.80%) in the study is similar to the previous studies in other countries [4, 12–14],but higher than that in the general population in China [15]. Additionally, the proportion of MRSA in *S. aureus* isolates was higher than the observed studies [4, 14].

In this study, we found that current cohabitation might be a risk factor for *S. aureus* carriage in drug users, which is different from another study [4]. One of the possible reasons might be that most drug users cohabitated with other drug users. This could provide more opportunities for sharing drugs [11]. HIV infection has been reported to be a risk factor for *S. aureus* carriage [16], however, we did not find any significance in this study. This could be caused by the limited number of drug users with HIV infection. Therefore, further studies need to be carried out to identify the risk factors for *S. aureus* carriage in drug users.

The patterns of antibiotic resistance on *S. aureus* isolates are consistent with limited available studies [8, 17, 18], with high proportions of penicillin, erythromycin, clindamycin and tetracycline resistance. The proportions of antibiotic resistance were higher in MRSA isolates than MSSA isolates, which is also observed in other studies [8, 18]. Teicoplanin has been widely used as an anti-MRSA agent in infectious patients in the past decades [19, 20], which can partially explain the high proportion of teicoplanin resistance in MRSA isolates. The most predominant MDR pattern of *S. aureus* isolates could partially demonstrate the high use of antibiotics in community-based drug users and provide evidence that healthcare workers need to be more careful with selection of antibiotics for drug users. Therefore, the administration of antibiotics for drug users should be strengthened.

The proportions of virulence genes were high in MRSA, suggesting the higher risks of MRSA isolates in causing virulence-related diseases, including Staphylococcal scalded skin syndrome, toxic shock syndrome, Staphylococcal food poisoning, etc. [21–23]. The proportions of virulence genes for MRSA isolates were higher than the observed studies [6, 24–26]. The results implied that drug users with MRSA carriage harboring virulence associated genes, might have higher risks for relevant disease and should draw more attention.

We found high proportions of ST5 and ST59 in this study and these STs were also globally reported in communities [27]. We also found hospital- (ST188) [28, 29] and livestock- (ST398) [30, 31] associated STs in this study. The results of CCs and STs for *S. aureus* isolates could demonstrate the multiple transmissions among human beings, livestock and environment, which are similar to previous studies [6, 24]. According to the results of SCC*mec* types, we could know the source of MRSA isolates might be both communities and healthcare settings, which is similar to the observed studies [4, 9]. Additionally, we found some *S. aureus* isolates displayed identical molecular characteristics, suggesting the possibility of cross-transmission between the communities and healthcare settings and this might be a potential risk for other populations. Relevant decolonization methods could be taken for drug users with MRSA carriage, which would help prevent further MRSA circulation [32].

Our study contributes to the understanding of the prevalence, risk factors, phenotypic and molecular characteristics for *S. aureus* carriage, particularly MRSA carriage, among drug users in China. Despite the strengths of this study, there are several limitations. First, it was a cross-sectional study. Thus, we could not determine the persistence of *S. aureus* carriage. Secondly, we only collected nasal swabs instead of nasopharyngeal swabs, which may lead to underestimation of the prevalence of *S. aureus* carriage. Thirdly, we did not collect information whether male participants were those who have sex with men due to confidentiality. We will explore it in future research. Finally, the generality of this study is limited owing to the small number of drug users.

## Conclusion

In summary, the prevalence of *S. aureus* nasal carriage was lower, while the prevalence of MRSA nasal carriage was moderate among community-based drug users but higher than that of general population in China. Cohabitation is a risk factor for *S. aureus* carriage. Phenotypic and molecular characteristics of MRSA isolates reveal serious antibiotic resistance, indicate the cross-circulation of MRSA isolates between communities and healthcare settings, and imply high opportunity of virulence-related diseases. Decolonization and antibiotic stewardship might be implemented for drug users with MRSA carriage, especially for those with risk factors.

## Data Availability

the data supporting the conclusions of this manuscript will be made available by the corresponding authors to any qualified researcher

## Abbreviations

aOR: Adjusted odds ratio
C: Chloramphenicol
CC: Clonal complex
CI: Confidence interval
CN: Gentamicin
DA: Clindamycin
E: Erythromycin
HIV: Human immunodeficiency virus
LZD: Linezolid
MDR: Multidrug resistance
MLST: Multilocus sequence typing
MRSA: Methicillin-resistant *S. aureus*
MSSA: Methicillin- sensitive *S. aureus*
MXF: Moxifloxacin
P: Penicillin
RD: Rifampin
*S. aureus*: *Staphylococcus aureus*
SCC*mec*: Staphylococcal cassette chromosome *mec*
SE: Staphylococcal enterotoxin
ST: Sequence type
SXT: Trimethoprim-sulfamethoxazole
TE: Tetracycline
TEC: Teicoplanin
